# Comparison of a twin interlocking derotation and compression screw cephalomedullary nail (InterTAN) with a single screw derotation cephalomedullary nail (proximal femoral nail antirotation): a systematic review and meta-analysis for intertrochanteric fractures

**DOI:** 10.1186/s13018-018-0749-6

**Published:** 2018-03-02

**Authors:** Leo Nherera, Paul Trueman, Alan Horner, Tracy Watson, Alan J. Johnstone

**Affiliations:** 1 Smith & Nephew Advanced Wound Management, Hull, UK; 2 Department of Orthopaedic Surgery, Saint Louis University School of Medicine, St. Louis, Missouri USA; 3 Orthopaedic Trauma Unit, Aberdeen Royal Infirmary, Aberdeen, UK

**Keywords:** Cephalomedullary nail, Intramedullary nails, Intertrochanteric hip fractures, Systematic review, Meta-analysis

## Abstract

**Background:**

Intertrochanteric hip fractures are common and devastating injuries especially for the elderly. Surgical treatment is the optimal strategy for managing intertrochanteric fractures as it allows early rehabilitation and functional recovery. The relative effects of internal fixation strategies for intertrochanteric fracture after operation remain limited to relatively small studies which create uncertainty in attempts to establish evidence-based best practice.

**Methods:**

We conducted a systematic review and meta-analysis of randomised controlled trials (RCTs) and observational studies to assess the clinical effectiveness of two commonly used intramedullary devices: a twin screw integrated cephalomedullary nail (InterTAN) versus a single screw cephalomedullary nail (proximal femoral nail antirotation) in patients with intertrochanteric fractures. The following outcomes were considered: revisions, implant-related failures, non-unions, pain, Harris Hip Score and intraoperative outcomes. Odds ratios or mean differences with 95% confidence intervals in brackets are reported.

**Results:**

Six studies met the inclusion criteria, two randomised controlled trials and four observational studies enrolling 970 patients with mean age of 77 years, and 64% of patients were female. There was a statistically significant difference (*p* value < 0.05) for revisions OR 0.27 (0.13 to 0.56), implant-related failures OR 0.16 (0.09 to 0.27) and proportion of patients complaining of pain OR 0.50 (0.34 to 0.74). There was no difference in non-unions and Harris Hip Score (*p* value > 0.05). There was a significant difference in blood loss and fluoroscopy usage in favour of PFNA, whilst no difference in operating times were observed between the two devices.

**Conclusions:**

Our meta-analysis suggests that a twin screw integrated cephalomedullary nail InterTAN is clinically more effective when compared to a single screw cephalomedullary nail proximal femoral nail antirotation resulting in fewer complications, fewer revisions and fewer patients complaining of pain. No difference has been established regarding non-unions and Harris Hip Score. Intraoperative outcomes favour PFNA with less blood loss and fluoroscopy usage. Further studies are warranted to explore the cost-effectiveness of these and other implants in managing patients with intertrochanteric fractures.

## Background

Intertrochanteric hip fractures are common and, often, devastating injuries especially for the elderly. By 2050, the annual number of hip fractures worldwide is estimated to surpass 6.3 million due to an ageing demographic in many Western countries. In the USA alone, the number of hip fractures is estimated to increase from about 320,000 per year to 580,000 by 2040. This increasing demand creates significant tension for the health service in terms of staff and resources required to manage these patients. In the USA, healthcare costs for the management of hip fractures are estimated to exceed $10 billion per year [1–6], whilst the impact on the UK health service is estimated to be $2 billion per year [7]. These costs are driven not only by the costs of the acute surgical procedure but also the post-acute care, including rehabilitation. Whilst hip fracture surgery is highly effective, patients are likely to experience significant morbidity in terms of pain, discomfort and limited mobility during their recovery and in many cases are unlikely to achieve pre-fracture levels of function [1, 4, 7]. Studies also suggest that there is an association between hip fracture and increased rates of mortality with 30% more deaths observed than the age-matched populations with and without hip fracture [7–13]. However, some caution should be taken in interpreting such data, as individuals who experience a hip fracture may be inherently more fragile and susceptible to ill-health.

Currently, intertrochanteric hip fractures are usually treated with intramedullary or extramedullary fixation devices. The known clinical benefits of internal fixation are rapid mobilisation, accelerated rehabilitation and, more importantly, significant pain relief [14, 15]. However, recent analyses have demonstrated that different devices suit different types of intertrochanteric fractures classified as stable/undisplaced fractures Orthopaedic Trauma Association (AO/OTA) classification A1 or displaced/unstable fractures AO/OTA classification A2 or A3 with the loss of the postero-medial buttress [16–18]. For stable fractures, fixation with a compression hip screws (CHS) has been shown to provide excellent clinical outcomes, whereas for unstable fractures, the use of intramedullary fixation devices have been shown to deliver superior clinical outcomes compared with CHS [16–18].

Two commonly used intramedullary fixation devices for displaced fractures are the proximal femoral nail anti-rotation (PFNA™) (Synthes, Solothurn, Switzerland) with a helical neck blade which provides rotational and angular stability and the TRIGEN^◊^ INTERTAN (Smith & Nephew, Memphis, Tennessee) which has a unique design of two cephalocervical screws that interlock and allow controlled linear intraoperative compression of the intertrochanteric fracture and subsequent rotational stability of the head and neck fragment [1, 14]. A number of studies have been conducted directly comparing these two devices to advise surgeons on device selection and best surgical practice [14, 19–23]; however, the findings of these studies are inconsistent, making it harder for surgeons to identify the ideal treatment option. To address this, we have performed a meta-analysis including all of the current evidence comparing the efficacy of InterTAN with the PFNA.

## Methods

### Data sources and searches

We searched the following electronic databases from January 2000 to February 2018: PubMed, Cochrane Database of Systematic Reviews (CDSR), Cochrane Central Register of Controlled Trials (CENTRAL), Health Technology Assessment (HTA) Database and ClinicalTrials.gov. The search terms used include the following: “hip fracture, reoperations, InterTAN, intertrochanteric fractures, Integrated 2 screw derotation cephalomedullary device, single screw cephalomedullary nail, proximal femoral nail anti-rotation, PFNA, PFNA-II”. To ensure completeness, we also used a pearl-growing technique, whereby the references of relevant papers identified in the original search were also searched.

### Study procedures

Two authors (LN and AH) independently screened all of the titles and abstracts based on the population, intervention, comparators and outcomes (PICO) framework [24] using a pilot-tested data extraction form. The quality of included RCTs was assessed using the Cochrane Collaboration’s risk of bias tool [25], and for observational studies, we used the Good Research for Comparative Effectiveness (GRACE) checklist [26].

### Study selection and eligibility criteria

We included prospective, randomised controlled trials (RCTs) and comparative observational studies with no language restriction if they enrolled participants diagnosed with intertrochanteric fractures and compared InterTAN with PFNA. We also included observational studies so as to utilise all of the existing evidence, an accepted technique that has been utilised by other researchers undertaking meta-analysis to assess other aspects of clinical care. We only considered full-text published studies without language restrictions. Studies were also included if they reported a minimum follow-up of 12 months. The pre-defined outcome measures of interest were functional measures (i.e. quality of life scores and pain), post-operative implant-related failures (i.e. cutout, varus collapse, shaft fractures) non-union, reoperation/revisions) and procedure measures (i.e. operative time, blood loss, fluoroscopy time). Following consultation with experienced clinicians, mortality was not included in this analysis. This was considered to be confounded by the nature of the patient group, i.e. most patients are elderly and frail and the clinicians indicated that the implants were unlikely to have impact upon mortality. The inclusion and exclusion criteria are outlined in Table 1.

**Table 1:** Inclusion exclusion criteria

Criteria	Inclusion	Exclusion
Type of study	RCTs, retrospective and prospective comparative observational studies	Systematic reviews, conference abstracts, case series, case reports, narrative reviews, editorials, opinions and studies performed in animals
Population	Adults with intertrochanteric hip fractures with subtrochanteric extension or subtrochanteric fractures	Stable fractures alone
Geographical location	Publications from any country	None
Interventions	Integrated 2 screw derotation cephalomedullary device (InterTAN)	Other nails other than InterTAN and PFNA. Less than 12 months follow-up
Comparators	Single screw cephalomedullary nail (PFNA)	Other nails other than InterTAN and PFNA. Less than 12 months follow-up
Outcomes of interest	Functional measures (i.e. quality of life scores and pain, Harris Hip Score) and post-operative implant-related failures (i.e. cutout, varus collapse, shaft fractures), non-union, reoperation/revisions and procedure measures (i.e. operative time, blood loss, fluoroscopy time)	

*RCT* randomised controlled trial, *PFNA* proximal femoral nail antirotation

### Data extraction

The following data from eligible studies was extracted: study characteristics (year of publication, sample size, country of study origin, length of follow-up), patient characteristics (gender, age), intervention/comparator and the pre-specified outcomes. Data extracted from each study is presented in Table 2

**Table 2:** Study characteristics of included studies

Study, year	Type of study and sample size	Mean age, years (range)	Percentage of males	Length of follow-up, months
Yu, 2016 [14]	Retrospective comparative *N* = 168 enrolled 147 available for analysis IT = 75 PFNA-II = 72	IT = 75.2 (66.4–84.0) PFNA-II = 74.2 (65.1–83.3)	IT = 35 PFNA-II = 32	12
Seyhan, 2015 [19]	RCT^a^ 75 (IT = 32; PFNA = 43)	IT = 75.3 (61.8–88.9) PFNA = 75.9 (62.2–89.6)	IT = 34.4 PFNA = 18.6	24
Zehir, 2015 [20]	Retrospective comparative *N* = 195 (IT = 102; PFNA = 93)	IT = 76.9 (70.2–83.6) PFNA = 77.2 (70.4–84.0)	IT = 38.2 PFNA = 38.5	16
Zhang, 2013 [21]	RCT^b^ *N* = 113 (IT = 47; PFNA-II = 46)	IT = 72.4 (64.8–80.0) PFNA-II = 72.4 (63.7–81.1)	IT = 40.4 PFNA-II = 33.9	12
Zhang, 2017 [22]	Retrospective comparative *N* = 174 (IT = 86; PFNA-II = 88)	IT = 72.7 (7.6) PFNA-II = 74.6 (6.3)	IT = 34.8 PFNA-II = 38.6	40
Zhang, 2017 [23]	Retrospective comparative *N* = 283 IT = 144 PFNA = 139	IT = 76.1 PFNA = 76.1	IT = 56 PFNA = 62	38.8

*IT* InterTAN, *PFNA* proximal femoral nail antirotation, *N* total number enrolled in the study

^a^Randomisation done by sealed envelopes

^b^Randomisation done by consecutive numbered and sealed envelopes based on a computer-generated list

### Meta-analysis

Meta-analyses were performed with a random effects model in Review Manager (RevMan), Version 5.3. Copenhagen: The Nordic Cochrane Centre, The Cochrane Collaboration, 2014. A standard pair-wise meta-analysis was conducted using either a fixed-effect or a random effects model depending upon the presence or absence of significant heterogeneity between studies. Heterogeneity of the included studies was assessed using the *I*^*2*^ statistic [27]. If the calculated *I*^*2*^ statistic was less than 50%, a fixed-effect model was used (no substantial heterogeneity), and when the calculated *I*^*2*^ statistic was more than 50%, a random effects model was used. For dichotomous outcomes, odds ratio (OR) was reported as the summary statistic, and for continuous outcomes, the (weighted) mean difference (MD) was reported. *p* values ≤ 0.05 were considered statistically significant.

Data were analysed separately for RCTs and observational studies. In a further analysis, all data were combined to ensure that all available evidence was utilised and in this paper we report the results of the combined analysis. A similar approach has been used successfully in other therapeutic areas such as cardiovascular medicine and wound care literature [28–30]. We performed sensitivity analyses by using alternative pooling methods (Peto method vs. Mantel–Haenszel method applicable to dichotomous data). Data was also analysed by study type, i.e. RCT and observational studies.

## Results

### Literature search

**Fig. 1:**
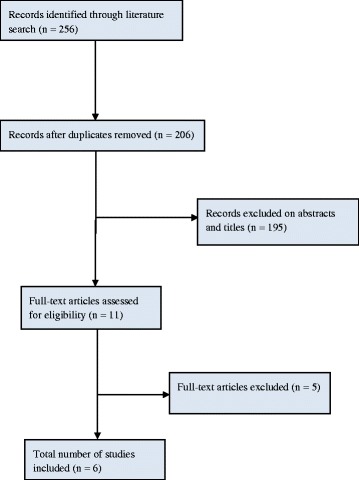
PRISMA flow diagram for studies included and excluded from the clinical effectiveness review

The electronic searches identified 256 articles, of which 50 were removed because they were either duplicates or unrelated. Eventually 26 articles were assessed for detailed evaluation. A careful screening of all titles excluded 15 articles leaving 11 studies for further full publication review. Six studies met the inclusion criteria and five did not because three were non-comparative and two were cadaver animal studies. Of those that met the inclusion criteria, two were RCTs and four were observational studies published between 2013 and 2017. A total of 970 patients (168 from RCTs and 802 from observational studies) were included in the analysis. Patients were equally distributed between InterTAN and PFNA, 486 and 484 respectively. Figure 1 summarises the flow diagram and the key characteristics of all included studies. All of the studies involved patients with intertrochanteric fractures and were followed up for at least 12 months. Three studies [14, 20, 21] only included patients with AO/OTA classification A2–A3 fractures, i.e. all unstable fractures, and the other three had mixed patients [19, 22, 23], 75% unstable and 25% had stable fractures between them respectively. The mean patient age in the included studies was 77 years, and 64% were females. All RCTs were rated as having an unclear risk of bias, generally due to a lack of information being reported in the methods. The majority of the observational studies were deemed to be of adequate quality according to the GRACE checklist [26].

### Clinical results

#### Revisions

**Fig. 2:**
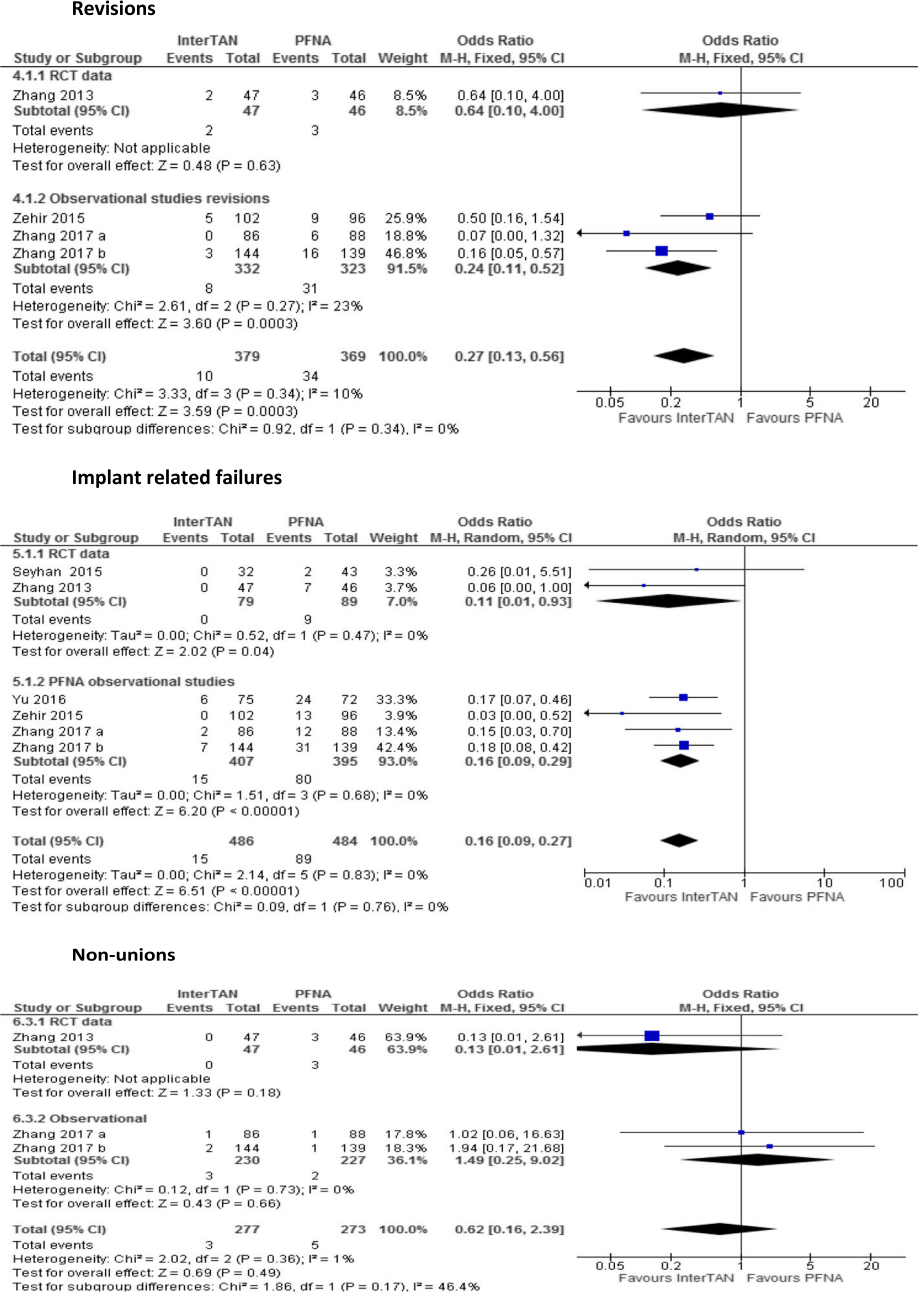
Impact of InterTAN compared with PFNA on revisions, implant-related failures (cutout, shaft fractures, varus collapse) and non-unions

Four studies reported on revisions, one RCT [21] and three observational studies [20, 22, 23], a total of 748 patients. Ten and 34 events were reported for InterTAN and PFNA groups respectively. There was a statistically significant difference in revision rates between InterTAN and PFNA (OR 0.27, 95% CI 0.13 to 0.56, *I*^*2*^ = 10%, *p* = 0.0003) (see Fig. 2).

### Implant-related failures

All six studies [14, 19–23] reported implant-related failure data defined as cutout, varus collapse and shaft fractures (*n* = 970 patients). A total of 15 and 89 events were reported for InterTAN and PFNA respectively. The pooled results showed that InterTAN was associated with a significantly reduced risk of implant failures by 84% compared to PFNA (OR 0.16, 95% CI 0.09 to 0.27, *I*^*2*^ = 0%, *p* = 0.00001). Five studies reported the outcome of shaft fracture [14, 19–23], five studies reported cutouts and shaft collapse whilst one study reported on varus collapse [14]. All of these outcomes were individually statistically significant in favour of InterTAN when compared to PFNA as shown in Fig. 2.

### Non-unions

Three studies reported on the incidence of non-unions: the RCT by Zhang [21] and two observational studies [22, 23]. The studies had 550 patients in total; three events were reported in the InterTAN group, and five events were reported in the PFNA group. There was no difference in non-union rates between InterTAN and PFNA (OR 0.62, 95% CI 0.16 to 2.39, *I*^*2*^ = 1%, *p* = 0.49) (see Fig. 2).

### Harris Hip Score

**Fig. 3:**
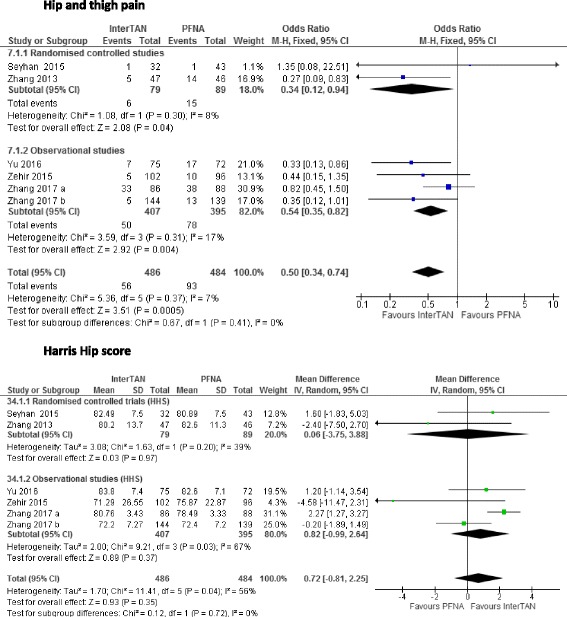
Impact of InterTAN compared with PFNA on the Harris Hip Score and the proportion of patients who complained of hip and thigh pain

All six studies [14, 19–23] reported on the HHS (*n* = 970 patients). Overall, there was no difference between patients treated with InterTAN compared to those treated with PFNA as shown by the pooled results using a random effects model due to evidence of statistical heterogeneity as shown by the *I*^*2*^ > 50% (MD 0.72, 95% CI − 0.81 to 2.25, *I*^*2*^ = 56%, *p* = 0.35) (see Fig. 3).

### Hip and thigh pain

All six studies [14, 19–23] reported on the proportions of patients who reported hip and thigh pain following the procedure (*n* = 970 patients). A total of 56 and 93 patients complained of pain after the operation for InterTAN and PFNA respectively. The pooled results showed that InterTAN significantly reduced the proportion of patients complaining of pain after the procedure by 50% compared to PFNA (OR 0.53, 95% CI 0.34 to 74, *I*^*2*^ = 7%, *p* = 0.00005) (see Fig. 3).

### Intraoperative outcomes (operating time, fluoroscopy time, blood loss) and other complications

**Fig. 4:**
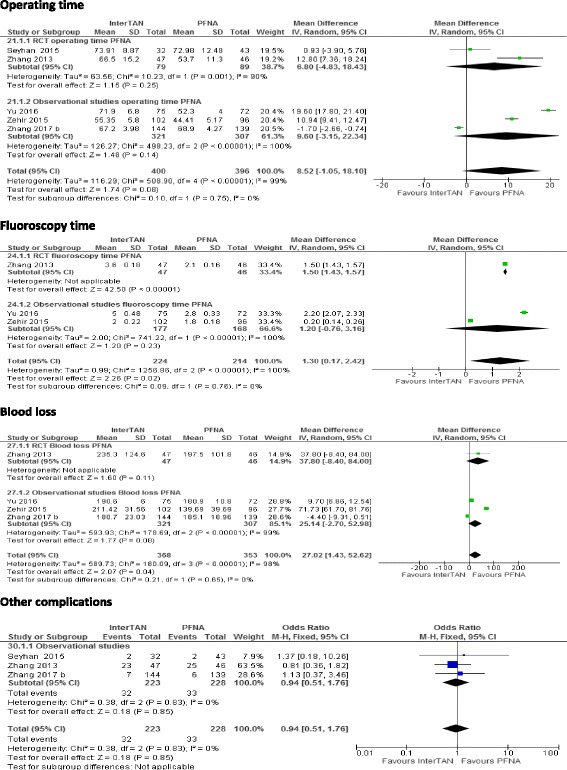
Impact of InterTAN compared with PFNA on operating time, fluoroscopy time, blood loss and other complications. Forest plots. The forest plots show the odds ratio (OR) calculated by the random effects model or the mean difference (MD) calculated by the fixed effects model. Squares represent individual study effects and diamonds represent the summary effect from the meta-analysis. Horizontal bars represent 95% CIs and the vertical line in plot is at 1 for OR and 0 for MD, corresponding to the null hypothesis of no effect. *I*^2^ = test of heterogeneity, CI confidence interval, df degree of freedom, M-H Mantel–Haenszel

Five studies reported on these outcomes [14, 19–21, 23] (a total of 796 patients). All the five studies reported on operating time, and there was a tendency of longer operating time with InterTAN. Overall, there was no difference in operating time between InterTAN and PFNA (MD 8.52, 95% CI −−1.05 to 18.10, *I*^*2*^ = 99%, *p* = 0.08). Three studies [14, 20, 21] reported on fluoroscopy time. There was longer fluoroscopy usage in the InterTAN group (MD 1.3, 95% CI 0.17 to 2.42, *I*^*2*^ = 100%, *p* = 0.02). Four studies [14, 20, 21, 23] reported on blood loss per case (in millilitres). There was a ststistically significant difference in blood loss, and more blood loss was recorded for InterTAN (MD 27.02, 95% CI 1.43 to 52.62, *I*^*2*^ = 99%, *p* = 0.04) (see Fig. 4).

### Other complications

Three studies (*n* = 451) reported on other complications [19, 21, 23]. The other complications reported consisted of deep vein thrombosis, cardiovascular disorders, pressure sores, urinary tract infection, pulmonary embolism and post-operative hematomas. In total, 32 and 33 complications were reported in InterTAN and PFNA respectively. Overall, there was no difference in the incidence of these complications between the two interventions (OR 0.94, 95% CI 0.46 to 1.90, *I*^*2*^ = 0%, *p* = 0.86; see Fig. 4).

### Sensitivity analysis

The sensitivity analyses using alternative analysis methods (Peto method vs. Mantel–Haenszel method), and considerations of heterogeneity (random effects vs. fixed effects) did not show important changes in the pooled effects for these outcomes except for Harris Hip Score. When a fixed effects model was used, the results of the analysis for HSS became statistically significant *p* = 0.0004. Study type did not change the overall conclusion. For instance, for the outcomes of implant-related failures and pain analysis, where there was a statistically significant difference between InterTAN and PFNA, RCT and observational evidence gave almost identical results as shown in Figs. 2 and 3. We also removed the studies by Seyhan [19] and Zhang [22, 23] which had mixed populations, i.e. 75% unstable patients and 25% stable between them from the analysis. Removing these studies resulted in the treatment effect increasing from 84 to 87% reduction in implanted-related failures.

## Discussion

The incidence of intertrochanteric fractures is rising due to a steady increase in life expectancy which in turn increases demand for surgery. With the emergence of value-based healthcare, there is a growing scrutiny of how best to provide high-quality care in a clinically and cost-effective manner, acknowledging limited healthcare budgets [1, 2]. Our study assessed the clinical performance of two most commonly used cephalomedullary nail devices for patients with unstable intertrochanteric fractures, InterTAN and PFNA, as outlined in our inclusion/exclusion criteria. We however accept that there are other devices which offer twin screw fixation such as the Aesculap Targon PFT® (B. Braun Hessen Germany) or the Orthofix VeroNail® (Orthofix, TX) as well as the other single screw devices such as Gamma 3 which were not the subject of our analysis. The findings from our meta-analysis confirm that InterTAN offers clinically and statistically significant benefits regarding revisions, long-term implant-related failures and post-operative pain compared to PFNA. No differences were observed between InterTAN and PFNA for non-unions and Harris Hip Score. For intraoperative outcomes, there was a difference in blood loss and fluoroscopy usage in favour of PFNA and there was no difference in operating times.

Unstable intertrochanteric fractures treated with cephalomedullary intramedullary devices are commonly associated with mild pain [14]. However, Yu et al. [14] proposed that long-term pain arises due to implant failures including lag screw cutout, shaft fractures or lateral protrusions of the distal end of the nail into the diaphysis [14]. In line with the proposed association between implant failure and long-term pain, our study demonstrated that the use of InterTAN resulted in a significant reduction in implant-related failures and reduced hip and thigh pain (50% fewer patients reported pain *p* = 0.0005). Although a direct causal relationship cannot be established from our analysis, it adds further weight to the conclusion by Yu et al. [14]. A potential explanation for the improved performance of InterTAN arises from its design which includes two integrated, interlocking lag screws that utilise a hybrid worm gear mechanism permitting better intraoperative fracture reduction and controlled compression of the intertrochanteric fracture. In addition, the trapezoidal proximal end of the nail may prevent uncontrolled shortening during fracture healing and limit varus collapse [1, 14]. Further analysis is required to substantiate the relationship between the mechanism of action and clinical outcomes.

The procedural outcomes identified that InterTAN was associated with a marginal increase in operative time (operation time and fluoroscopy usage) as well as an increase in blood loss. Although the studies did not identify the causes of this, differences in operative techniques associated with each device may explain this. For example, the trapezoidal proximal end of the InterTAN device may require additional reaming of the intramedullary canal, which can result in extended operative and fluoroscopy time. However, the differences seen in the study are marginal when considered in the context of the entire procedure. Similarly, marginal differences were observed in blood loss, which is most likely an association with the longer surgical time associated with InterTAN. Nonetheless, Zhang et al. [22] cautioned against choosing implants based on these parameters as they are likely to be influenced by other factors, instead preferring to base such decisions on the long-term efficacy of implants.

One factor that impacts complication rates, especially of intramedullary nail implants, is the dimension of the nail, i.e. long or short nails [22, 31–33]. In four out of the six studies, nail dimensions were reported and the range for InterTAN was 18–20 cm whilst PFNA was 20 to 28 cm which are deemed to be short nails. Zhang [22] and Li [31] noted that the use of long PFNA nails in patients improved the clinical outcomes compared to short nails especially failure rates and pain. Other authors that have looked at long and short PFNA nails and found no difference in clinical outcomes except for intraoperative outcomes such as operating time and blood loss which favour the smaller nails [32–34]. Although our meta-analysis found significant differences in clinical outcomes between InterTAN and PFNA, we cannot be certain that nail dimension did not contribute to the outcomes. Further research on the relationship between nail length and outcomes would be beneficial.

The study attempted to employ innovative techniques to consider all available relevant data on the performance of InterTAN and PFNA. Systematic reviewing has typically been constrained to the use of randomised controlled trials on the basis that this study design minimises any potential for bias. However, it should be acknowledged that in doing so, RCTs often limit their external validity by applying strict inclusion/exclusion criteria and creating an environment that may not be reflective of typical practice. Whilst observational studies are potentially subject to more bias, they do provide useful insights into product performance in real-world practice settings. Furthermore, in the case of surgical interventions where RCTs are often small in size, observational studies can often provide far larger samples, as is the case with the current study. Methods that can combine these two sources of data remain relatively immature, although there is an increasing body of evidence that has sought to do so in order to make the best use of all available data to inform treatment decisions.

Any uncertainty in the appropriateness of combining datasets can be addressed by considering the different sources of data separately, seeking consistency and running sensitivity analyses based on the single sources of data. In the current study, both the RCT and observational evidence consistently suggested that patients treated with InterTAN have a significantly lower risk of complications than those treated with PFNA, including the risk of revisions, cutout, varus collapse and shaft fractures. The consistency of findings across the studies and the relatively large magnitude of effect, i.e. 73% and 84% reduction in revisions and post-operative complications respectively, increase the credibility of our findings.

There are limitations associated with this study. The trials we included in the analysis suffered from some methodological limitations, as do many other surgical trials. For instance, most of the RCTs included in our analyses had small patient numbers whilst the observational studies, as expected, had bigger patient numbers. This may have resulted in an imprecise estimation of effects from RCTs. We also noted that two of the studies [19, 22] included both stable and unstable fractures although the majority of fractures were unstable. To complicate things further, the results were not reported according to the stability of the fracture. Clearly, we would have liked the results to have been reported according to fracture stability to be certain which sub-group of patients benefit most from the interventions that they received. We therefore were unable to explore if the treatment effects were influenced by fracture stability in this particular study. Nonetheless when these studies were removed from the analysis in sensitivity analysis, the results remained statistically significant with the treatment effect improving slightly from 84% reduction in implant failures to 87% *p* = 0.0001.

## Conclusion

In conclusion, the current body of evidence suggests that the use of InterTAN compared to the PFNA in treating patients with unstable intertrochanteric fractures results in clinical significant reductions in revisions, the proportion of patients complaining of pain after the surgery and post-operative implant-related complications. There were no differences on non-unions and Harris Hip score. There was less fluoroscopy usage and less blood loss with PFNA. Further research on the cost-effectiveness of these implants would provide further information to ensure that treatment decisions are both clinically and cost-effective.

## Abbreviations

AO/OTA: Orthopaedic Trauma Association
CDSR: Cochrane Database of Systematic Reviews
CENTRAL: Cochrane Register of Controlled Trials
CHS: Compression hip screws
CI: Confidence interval
GRACE: Good Research for Comparative Effectiveness
HHS: Harris Hip Score
HTA: Health technology assessment
MD: Mean difference
OR: Odds ratio
PFNA: Proximal femoral nail anti-rotation
RCT: Randomised controlled trials
SMD: Standardised mean difference

## References

[1] Ruecker AH, Rupprecht M, Gruber M, Gebauer M, Barvencik F, Briem D (2009). The treatment of intertrochanteric fractures: results using an intramedullary nail with integrated cephalocervical screws and linear compression. J Orthop Trauma.

[2] Mundi S, Pindiprolu B, Simunovic N, Bhandari M (2014). Similar mortality rates in hip fracture patients over the past 31 years. Acta Orthop.

[3] De Laet CE, van Hout BA, Burger H, Hofman A, Pols HA (1997). Bone density and risk of hip fracture in men and women: cross sectional analysis. BMJ.

[4] Burge R, Dawson-Hughes B, Solomon DH, Wong JB, King A, Tosteson A (2007). Incidence and economic burden of osteoporosis-related fractures in the United States, 2005-2025. J Bone Miner Res.

[5] Søgaard AJ, Holvik K, Meyer HE, Tell GS, Gjesdal CG, Emaus N, Grimnes G, Schei B, Forsmo S, Omsland TK (2016). Continued decline in hip fracture incidence in Norway: a NOREPOS study. Osteoporos Int.

[6] Nieves JW, Bilezikian JP, Lane JM, Einhorn TA, Wang Y, Steinbuch M, Cosman F (2010). Fragility fractures of the hip and femur: incidence and patient characteristics. Osteoporos Int.

[7] Lisk R, Yeong K. Reducing mortality from hip fractures: a systematic quality improvement programme. BMJ Quality Improvement Reports. 2014; 10.1136/bmjquality.u205006.w2103. 10.1136/bmjquality.u205006.w2103 PMC4949608 27493729

[8] Amphansap T, Nitiwarangkul L (2015). One-year mortality rate after osteoporotic hip fractures and associated risk factors in Police General Hospital. Osteoporosis and Sarcopenia.

[9] Klop C, Welsing PM, Cooper C, Harvey NC, Elders PJ, Bijlsma JW, Leufkens HG, de Vries F. Mortality in British hip fracture patients, 2000-2010: a population-based retrospective cohort study. Bone. 2014; 10.1016/j.bone.2014.06.011. 10.1016/j.bone.2014.06.011 24933345

[10] Imai N, Endo N, Hoshino T, Suda K, Miyasaka D, Ito T (2016). Mortality after hip fracture with vertebral compression fracture is poor. J Bone and Mineral Metabolism.

[11] Li S, Sun T, Liu Z (2016). Excess mortality of 1 year in elderly hip fracture patients compared with the general population in Beijing, China. Arch Osteoporos.

[12] Papadimitriou N, Tsilidis KK, Orfanos P, Benetou V, Ntzani EE, Soerjomataram I (2017). Burden of hip fracture using disability-adjusted life-years: a pooled analysis of prospective cohorts in the CHANCES consortium. The Lancet Public Health.

[13] Songpatanasilp T, Sritara C, Kittisomprayoonkul W, Chaiumnuay S, Nimitphong H (2016). Thai Osteoporosis Foundation (TOPF) position statements on management of osteoporosis. Osteoporosis and Sarcopenia.

[14] Yu J, Zhang C, Li L, Kwong JSW, Xue L, Zeng X, Tang L, Li Y, Sunb X. Internal fixation treatments for intertrochanteric fracture: a systematic review and meta-analysis of randomized evidence. Sci Rep. 2015; 10.1038/srep18195. 10.1038/srep18195 PMC4676068 26657600

[15] Queally JM, Harris E, Handoll HHG, Parker MJ. Intramedullary nails for extracapsular hip fractures in adults. Cochrane Database Syst Rev. 2014; 10.1002/14651858.CD004961.pub4. 10.1002/14651858.CD004961.pub4 PMC10835205 25212485

[16] Matre K, Havelin LI, Gjertsen JE, Espehaug B, Fevang JM (2013). Intramedullary nails result in more reoperations than sliding hip screws in two-part intertrochanteric fractures. Clin Orthop Relat Res.

[17] Matre K, Havelin LI, Gjertsen JE, Vinje T, Espehaug B, Fevang JM (2013). Sliding hip screw versus IM nail in reverse oblique trochanteric and subtrochanteric fractures. A study of 2716 patients in the Norwegian Hip Fracture Register. Injury.

[18] Matre K, Vinje T, Havelin LI, Gjertsen JE, Furnes O, Espehaug B, Kjellevold SH, Fevang JM (2013). TRIGEN INTERTAN intramedullary nail versus sliding hip screw: a prospective, randomized multicenter study on pain, function, and complications in 684 patients with an intertrochanteric or subtrochanteric fracture and one year of follow-up. J Bone Joint Surg Am.

[19] Seyhan M, Turkmen I, Unay K, Ozkut AT (2015). Do PFNA devices and Intertan nails both have the same effects in the treatment of trochanteric fractures? A prospective clinical study. J Orthop Sci.

[20] Zehir S, Şahin E, Zehir R (2015). Comparison of clinical outcomes with three different intramedullary nailing devices in the treatment of unstable trochanteric fractures. Ulus Travma Acil Cerrahi Derg.

[21] Zhang S, Zhang K, Jia Y, Yu B, Feng W (2013). InterTan nail versus proximal femoral nail antirotation-Asia in the treatment of unstable trochanteric fractures. Orthopedics.

[22] Zhang H, Zeng X, Zhang N, Zeng D, Xu P, Zhang L, Chen D, Yu W, Zhang X. INTERTAN nail versus proximal femoral nail antirotation-Asia for intertrochanteric femur fractures in elderly patients with primary osteoporosis. J Int Med Res. 2017; 10.1177/0300060517710584. 10.1177/0300060517710584 PMC5625524 28587540

[23] Zhang H, Zhu X, Pei G, Zeng X, Zhang N, Xu P, Chen D, Yu W, Zhang X. A retrospective analysis of the InterTan nail and proximal femoral nail anti-rotation in the treatment of intertrochanteric fractures in elderly patients with osteoporosis: a minimum follow-up of 3 years. Orthop Surg Res. 2017; 10.1186/s13018-017-0648-2 10.1186/s13018-017-0648-2 PMC5634834 29017580

[24] Shamseer L, Moher D, Clarke M, Ghersi D, Liberati A, Petticrew M, et al. Preferred reporting items for systematic review and meta-analysis protocols (PRISMA-P) 2015: elaboration and explanation. BMJ. 2015; 10.1136/bmj.g7647. 10.1136/bmj.g7647 25555855

[25] Higgins JP, Altman DG, Gotzsche PC, Juni P, Moher D, Oxman AD, et al. The Cochrane Collaboration’s tool for assessing risk of bias in randomised trials. BMJ. 2011; 10.1136/bmj.d5928. 10.1136/bmj.d5928 PMC3196245 22008217

[26] Dreyer NA, Velentgas P, Westrich K, Dubois R (2014). The GRACE checklist for rating the quality of observational studies of comparative effectiveness: a tale of hope and caution. J Manag Care Spec Pharm.

[27] Higgins JPT, Thompson SG (2002). Quantifying heterogeneity in a meta-analysis. Statist Med.

[28] Bonovas S, Filioussi K, Tsavaris N, Sitaras NM (2005). Use of statins and breast cancer: a meta-analysis of seven randomized clinical trials and nine observational studies. J Clin Oncol.

[29] Nherera LM, Trueman P, Roberts CD, Berg L (2017). Silver delivery approaches in the management of partial thickness burns: a systematic review and indirect treatment comparison. Wound Rep Reg.

[30] Shrier I, Boivin JF, Steele RJ, Platt RW, Furlan A, Kakuma R (2007). Should meta-analyses of interventions include observational studies in addition to randomized controlled trials? A critical examination of underlying principles. Am J Epidemiol.

[31] Li Z, Liu Y, Liang Y, Zhao C, Zhang Y (2015). Short versus long intramedullary nails for the treatment of intertrochanteric hip fractures in patients older than 65 years. Int J Clin Exp Med.

[32] Raval P, Ramasamy A, Raza H, Khan K, Awan N (2016). Comparison of short vs long anti-rotation in treating trochanteric fractures. Malaysian Orthopaedic Journal.

[33] Hong CC, Nashi N, Makandura MC, Tan JHJ, Peter L, Murphy D (2017). The long and short of cephalomedullary nails in the treatment of osteoporotic pertrochanteric fracture. Singap Med J.

[34] Dunn J, Kusnezov N, Bader J (2016). Long versus short cephalomedullary nail for trochanteric femur fractures (OTA 31-A1, A2 and A3): a systematic review. J Orthop Traumatol.

